# Ionizing radiation modifies immune-related molecular profiles of tumor-derived exosomes

**DOI:** 10.1186/2051-1426-3-S2-P382

**Published:** 2015-11-04

**Authors:** Julie M Diamond, Jessica R Chapman, Beatrix Ueberheide, Silvia Formenti, Sandra Demaria

**Affiliations:** 1 NYU School of Medicine, Pathology Department, New York, NY, USA; 2 NYU School of Medicine, Proteomics Resource Center, New York, NY, USA; 3 Weill Cornell Medical College, Radiation Oncology Department, New York, NY, USA

## Background

Exosomes are microvesicles (30-100nm) released from living cells that shuttle and transfer selected cellular biomolecules, including cytokines, cell surface molecules, growth factors, mRNA, and miRNA. Tumor-derived exosomes (TEX) allow for a sophisticated means of communication with a variety of cells, including immune cells, within the tumor microenvironment. Ionizing radiotherapy (RT) promotes anti-tumor immune responses by promoting uptake of tumor antigens by dendritic cells and by enhancing antigen presentation to activate effector T cells. We hypothesized that TEX released from irradiated tumors may play a role in altering the susceptibility of tumor cells to immune-mediated rejection.

## Methods

Mouse mammary carcinoma cells TSA were treated *in vitro* with sham RT, 1 dose of 20Gy, or 3 fractions of 8Gy (8Gyx3). Cells were transferred to exosome-depleted media following RT and supernatant was collected 48hr later. TEX were isolated using differential ultracentrifugation and purified by sucrose gradient. Electron microscopy confirmed TEX expected size and morphology. TEX were lysed for protein identification using label-free quantitation mass spectrometry (LFQ-MS) followed by MS/MS analyses. To characterize miRNA signatures of TEX and their parent cells, RNA was isolated for nanoString nCounter Mouse miRNA expression assay kit using a panel of 578 mouse miRNAs. Normalized results were analyzed with MultiExperiment Viewer.

## Results

LFQ-MS revealed significant changes in TEX proteomic profiles when their parent cells were treated with RT. Significant differences in TEX protein composition was observed based on the RT regimen used (20Gy vs 8Gyx3). In two separate experiments, TEX from 8Gyx3-but not 20Gy-treated TSA cells showed significant increase in proteins involved in the Antigen Processing and Presentation pathway (p= 0.012). Additionally, 17 unique proteins were present in TEX from 8Gyx3-treated TSA cells. Among them were proteins involved in T cell development, MHC class I peptide processing, and proinflammatory lipid signaling, which were not present in TEX from 20Gy-treated cells. Fractionated radiation induced downregulation of 73% of miRNAs expressed in untreated TSA cells. Unique miRNA expression patterns emerged in TEX, which were RT regimen-dependent.

## Conclusions

Data indicate that cancer cell irradiation alters the molecular composition of released TEX, with some changes being RT regimen-dependent. Changes in immune-related pathways were induced by 8Gyx3 but not 20Gy RT, suggesting that TEX could be biomarkers for more pro-immunogenic RT regimens (Dewan et al, Clin Cancer Res, 2009). We are currently investigating the contribution of TEX to RT-induced T cell priming. TEX-mediated communication networks may provide new therapeutic targets to improve responses to cancer immunotherapy.

